# Current Evidence on the Treatment of Median Arcuate Ligament Syndrome: A Comprehensive Review

**DOI:** 10.7759/cureus.113409

**Published:** 2026-07-26

**Authors:** Kalliopi-Kleio E Gotti, Maria D Velikoudi, Charis G Kourgiali, Dimitrios A Chatzelas

**Affiliations:** 1 2nd Department of Surgery, “G. Gennimatas” General Hospital, Faculty of Medicine, Aristotle University of Thessaloniki, Thessaloniki, GRC

**Keywords:** celiac artery compression, dunbar syndrome, laparoscopic surgery, median arcuate ligament syndrome, robotic surgery

## Abstract

Median arcuate ligament syndrome (MALS) is an uncommon and controversial disorder characterized by chronic abdominal symptoms associated with extrinsic compression of the celiac artery (CA) and adjacent celiac plexus by the median arcuate ligament (MAL). Although anatomical CA compression is relatively common, only a small proportion of individuals develop clinically significant disease, making accurate diagnosis and patient selection fundamental to successful treatment. This narrative review critically evaluates the current evidence on the management of MALS, focusing on patient selection, the role of celiac plexus blockade, operative decompression techniques, adjunctive revascularization, treatment outcomes, and postoperative surveillance. A comprehensive literature search of PubMed/MEDLINE, Scopus, Web of Science, and the Cochrane Library was performed up to June 2026, with an emphasis on clinical guidelines, systematic reviews, multicenter studies, and comparative series. Contemporary evidence supports a multidisciplinary, exclusion-based diagnostic approach, integrating compatible symptoms with dynamic vascular imaging, while recognizing that radiographic CA compression alone is insufficient to establish the diagnosis. Surgical MAL release, often combined with celiac plexus neurolysis, remains the cornerstone of treatment, with minimally invasive approaches generally favored because of lower perioperative morbidity and faster recovery. Primary endovascular treatment without ligament release is not recommended, whereas staged revascularization should be reserved for selected patients with persistent symptoms and objectively significant residual stenosis after adequate decompression. Despite encouraging outcomes in carefully selected patients, the available evidence is limited by retrospective study designs, heterogeneous diagnostic criteria, inconsistent outcome reporting, and a lack of randomized controlled trials. Future prospective studies and standardized reporting are required to optimize patient selection and establish evidence-based treatment algorithms.

## Introduction and background

Median arcuate ligament syndrome (MALS), also termed celiac artery (CA) compression syndrome or Dunbar syndrome, describes the coexistence of otherwise unexplained abdominal symptoms and extrinsic compression of the proximal CA by the median arcuate ligament (MAL) [1,2]. The MAL is a fibrous arch connecting the diaphragmatic crura anterior to the aortic hiatus. In individuals with a relatively low MAL or high CA origin, the ligament may cross anterior to the proximal CA and produce dynamic compression, typically accentuated during expiration [1-4].

Anatomic compression is substantially more common than clinically significant MALS, which is rare and accounts for only a small proportion of patients undergoing evaluation for chronic abdominal pain. Characteristic celiac indentation may be found incidentally on cross-sectional imaging in asymptomatic individuals, making it inappropriate to diagnose MALS from imaging alone [3,5]. The distinction between an anatomic phenomenon and a clinical syndrome is fundamental; intervention directed at an incidental radiologic finding exposes patients to operative morbidity without a predictable clinical benefit [2].

The classical clinical presentation comprises chronic postprandial epigastric pain, reduced food intake or food avoidance, nausea, vomiting, and weight loss. Some patients report exercise-induced pain, diarrhea, or bloating. Women predominate, and many patients have experienced symptoms for years before referral [6-9]. Nevertheless, no individual symptom, physical finding, or imaging feature is sufficiently specific to establish the diagnosis.

Treatment remains controversial despite the increasing adoption of minimally invasive techniques, because the underlying pathophysiology is uncertain, diagnostic criteria have varied markedly, and most outcome evidence consists of retrospective series. The vascular hypothesis attributes symptoms to reduced celiac perfusion, impaired postprandial hyperemia, or diversion of superior mesenteric artery flow through pancreaticoduodenal collaterals. The neurogenic hypothesis attributes symptoms to compression and irritation of the celiac ganglion and plexus, with altered visceral nociception, sympathetic activation, vasoconstriction, or gastrointestinal dysmotility [6,10-13]. The frequent improvement after ganglionectomy or neurolysis, despite persistent arterial stenosis, and the response of some patients to celiac plexus blockade support an important neural component [6,14,15]. However, these mechanisms are not mutually exclusive.

The objective of this narrative review is to critically evaluate and synthesize the current evidence on the treatment of MALS, with emphasis on appropriate patient selection, the role of celiac plexus blockade, operative decompression techniques, and the indications for adjunctive CA revascularization. The review also aims to compare the outcomes and limitations of open, laparoscopic, and robot-assisted approaches; examine factors associated with treatment success and recurrence; and place contemporary practice within the framework of a practical evidence-based management approach.

## Review

Methods

A narrative literature review was performed using PubMed/MEDLINE, Scopus, Web of Science, and the Cochrane Library up to June 2026. Search terms included “median arcuate ligament syndrome,” “celiac artery compression,” “Dunbar syndrome,” “treatment,” “median arcuate ligament release,” “celiac ganglionectomy,” “laparoscopic,” “robotic,” “open surgery,” “celiac plexus block,” “angioplasty,” “stent,” “revascularization,” “outcomes,” and “recurrence.” The reference lists of relevant articles were screened for additional sources. Only articles published in English and indexed in peer-reviewed journals were included. Priority was given to systematic reviews, multicenter studies, comparative series, prospective cohorts, and larger case series. Earlier landmark studies were retained, when necessary, to describe the development of operative treatment or when they provided long-term data that are not available from contemporary series. Case reports were used selectively to discuss unusual complications or adjunctive interventions and were not used to estimate effectiveness. Due to heterogeneity in diagnostic definitions, interventions, follow-up, and outcome reporting, no quantitative analysis was performed.

Patient Selection for Treatment

Treatment decisions begin with recognition that celiac compression is not equivalent to MALS. The diagnosis requires a compatible symptom complex, exclusion of more prevalent gastrointestinal and vascular disorders, and demonstration of dynamic external compression [8]. Depending on the presentation, the differential diagnosis includes peptic ulcer disease, biliary disease, chronic pancreatitis, gastroparesis, inflammatory bowel disease, celiac disease, functional gastrointestinal disorders, and chronic atherosclerotic mesenteric ischemia [8,16,17].

The European Society for Vascular Surgery guidelines propose a relatively restrictive clinical definition: 1. postprandial pain accompanied by dietary modification, unexplained weight loss, or unexplained diarrhea; 2. at least 70% CA stenosis from external compression, demonstrated by two imaging techniques, with inspiratory and expiratory thin-section computed tomography angiography (CTA) included among the imaging assessments; and 3. no other abnormality on abdominal USG or upper gastrointestinal endoscopy [16]. These criteria are more stringent than those used in many historical series and are intended to reduce overtreatment of incidental compression.

Duplex USG during inspiration and expiration is recommended as first-line vascular imaging. Findings supporting dynamic compression include elevated expiratory peak systolic velocity, reduction in velocity during deep inspiration or standing, focal aliasing, post-stenotic dilatation, and marked deflection of the proximal CA [18,19]. CTA demonstrates focal superior indentation, the characteristic hooked configuration, post-stenotic dilatation, and collateral vessels, while excluding atherosclerosis and other intra-abdominal disease [2,3]. Magnetic resonance angiography (MRA) can be used when avoidance of radiation or iodinated contrast is desirable. Conventional angiography is now generally reserved for unresolved diagnostic questions, pressure-gradient assessment, or planned endovascular treatment [16].

No imaging threshold reliably predicts postoperative symptom relief. Patel MV et al. found that conventional imaging characteristics were unable to discriminate patients who would respond to laparoscopic release [20]. Likewise, postoperative symptom improvement may occur despite residual arterial stenosis, whereas complete normalization of flow does not guarantee clinical success [6]. Imaging should therefore confirm the relevant anatomy but should not be used as the sole indication for treatment. An exception may arise when celiac compression is associated with clinically significant pancreaticoduodenal collateral aneurysms, but management in that setting is directed primarily at aneurysm rupture risk rather than symptoms and must be individualized [10].

Evaluation should include vascular or general surgeons, gastroenterologists, radiologists experienced in dynamic mesenteric imaging, pain specialists, and, when appropriate, dietitians and mental health professionals. Psychiatric diagnoses and functional pain disorders are relatively common in referred MALS populations. Their presence does not establish that symptoms are psychogenic and should not automatically preclude surgery; however, psychiatric comorbidity has been associated with poorer patient-reported quality-of-life outcomes in some cohorts [21,22].

Autonomic dysfunction, postural orthostatic tachycardia syndrome, hypermobility disorders, gastroparesis, and other chronic pain syndromes are also reported in MALS populations. These conditions may coexist with true CA compression but can produce persistent symptoms after anatomically successful release [8-10,12,22]. Preoperative counselling should distinguish the symptoms most plausibly attributable to MALS from symptoms likely to persist because of comorbidities.

Conservative Treatment

There is no established pharmacological therapy that can reverse the underlying compression. Analgesics, anti-emetics, dietary modification, nutritional support, treatment of gastroparesis, and management of coexisting gastrointestinal or psychiatric disease may provide symptomatic benefit but are not definitive treatment for appropriately diagnosed MALS [2,8,16]. Nutritional optimization is important in patients with severe weight loss because malnutrition can increase perioperative risk and prolong recovery. Observation is appropriate for asymptomatic CA compression and for patients whose symptoms are atypical, mild, or better explained by another disorder [16,17].

Celiac Plexus Block

Temporary celiac plexus blockade has gained attention as a diagnostic enrichment tool. Local anesthetic is delivered to the celiac plexus by percutaneous, fluoroscopic, CT-guided, or endoscopic ultrasound-guided techniques. A marked but temporary reduction in typical pain supports a neurogenic component and may identify patients more likely to improve after surgical neurolysis [14,15]. In the multicenter cohort reported by DeCarlo C et al., absence of symptom relief after preoperative celiac plexus blockade was associated with a higher risk of treatment failure [22]. In a subsequent series of 28 block-responsive patients selected for operative release, 27 experienced immediate postoperative improvement, although mean follow-up was only approximately three months [15]. Another contemporary study found substantial immediate pain relief after temporary blockade in patients investigated for neurogenic MALS [23].

These findings are encouraging but do not establish the block as a definitive diagnostic test. Protocols vary in anesthetic agent, imaging guidance, response threshold, and duration of assessment. Placebo effects are particularly relevant in chronic pain populations. A positive block may enrich surgical selection, but a negative block should be interpreted cautiously, especially when technical adequacy is uncertain [14,23]. Prospective studies reporting block results and long-term outcomes are required.

Surgical Decompression

Principles of operative treatment: The central therapeutic principle is complete division of the MAL and constricting diaphragmatic fibers from the anterior and lateral surfaces of the CA origin. Most contemporary surgeons also perform extensive removal or division of the surrounding celiac plexus and ganglionic tissue. This combined decompression and neurolysis addresses both proposed vascular and neurogenic mechanisms [7,10,24]. The operation should expose the proximal celiac trunk and the origins of the common hepatic, splenic, and left gastric arteries sufficiently to confirm complete release [7]. Intraoperative duplex USG, direct pressure measurements, indocyanine green assessment, or completion angiography can document improved flow, but evidence is limited [16]. The proximity of the abdominal aorta, CA, hepatic and splenic branches, inferior phrenic vessels, pancreas, and diaphragmatic crura makes experience with upper retroperitoneal vascular anatomy essential.

Open release: Open decompression was historically performed through an upper midline, transverse, or thoracoabdominal incision. It permits direct control of the celiac origin and enables simultaneous endarterectomy, patch angioplasty, reimplantation, or bypass when fixed arterial disease is present [7,10,24]. Long-term open series demonstrated symptom improvement in approximately 55-75%, sometimes with better results when arterial reconstruction accompanied decompression [25,26].

The disadvantages are greater postoperative pain, longer hospitalization, slower return to oral intake, incisional morbidity, and the physiological burden of laparotomy. Open release remains relevant when minimally invasive exposure is unsafe, major bleeding requires conversion, prior operations make laparoscopy difficult, or concomitant arterial reconstruction is clearly indicated [7,10,24].

Laparoscopic release: Laparoscopic release has become the most commonly reported contemporary approach. The lesser sac or gastrohepatic region is entered, the left gastric or common hepatic artery is identified, and dissection proceeds proximally to the celiac origin and abdominal aorta. The diaphragmatic fibers are divided until the celiac origin is visibly free, accompanied by circumferential or near-circumferential neurolysis [20,24].

Laparoscopy is associated with shorter hospitalization, earlier oral intake, lower wound morbidity, and faster recovery than open surgery [27,28]. The review by Jimenez JC et al. included 400 surgically treated patients and reported immediate symptom improvement in 85%. Conversion to open surgery occurred in 9.1% of laparoscopic procedures, principally because of bleeding. Late recurrence was reported in 6.8% after open treatment and 5.7% after laparoscopic treatment, although follow-up and definitions varied substantially [27].

Subsequent evidence has been less optimistic regarding durability. Metz FM et al. reviewed 38 adult studies, including 880 patients, and six pediatric studies, including 195 patients. Most studies reported durable improvement exceeding 70%, but heterogeneity, inconsistent diagnostic criteria, incomplete follow-up, and high risk of bias precluded formal meta-analysis [29]. Thus, the often-quoted 70-85% success rate should be presented as an approximate range from selected observational cohorts rather than as a precise treatment effect.

Robotic-assisted release: Robotic assistance provides articulated instruments, three-dimensional visualization, tremor filtration, and improved suturing ergonomics. These features may facilitate dissection around the proximal CA and management of small vascular injuries. Published robotic series generally report high technical success and short hospital stays [30,31]. However, comparative evidence has not demonstrated consistent clinical superiority. Do MV et al. found that conventional laparoscopy had a shorter operative time than robotic-assisted surgery, with no clear difference in clinical outcome [30]. More recent retrospective comparisons have produced variable findings regarding blood loss, operative time, recurrence, and symptom improvement [31,32]. Selection bias, surgeon learning curves, small cohorts, and different definitions of recurrence limit interpretation.

A systematic review and meta-analysis comparing laparoscopic and robotic approaches reported broadly favorable perioperative outcomes for both techniques, but the evidence remained derived from non-randomized studies and did not establish a decisive long-term advantage for either platform [33]. The operative principle of complete decompression and neurolysis is probably more important than the choice between laparoscopic and robotic instrumentation [30].

Retroperitoneal endoscopic release: Video-assisted retroperitoneal endoscopic release approaches the celiac origin without entering the peritoneal cavity. van Petersen AS et al. reported this technique in 46 patients; approximately 89% had some symptomatic improvement, and 65% became symptom-free, with a low conversion rate [34]. Potential advantages include direct access to the abdominal aorta and avoidance of intraperitoneal adhesions. However, the retroperitoneal working space is restricted, orientation can be difficult, and adoption has remained limited to specialized centers [34].

Role of CA Revascularization

Primary endovascular treatment: Angioplasty or stenting without prior ligament release does not address the external mechanical compression. Repeated respiratory motion, celiac angulation, elastic recoil, and direct contact with the ligament may produce residual stenosis, early restenosis, stent deformation, fracture, or migration [16,35,36]. Consequently, primary endovascular therapy is not recommended for uncomplicated MALS [16]. This is biologically plausible and supported by failed angioplasty experiences and cases of stent-related mechanical complications, but not by randomized data [35,36].

Secondary revascularization: Residual fixed stenosis can persist after complete MAL division because of intimal hyperplasia, chronic arterial remodeling, fibrosis, dissection, or established occlusion. Revascularization may be considered when all of the following are present: technically adequate release, persistent or recurrent symptoms, objectively important residual celiac stenosis or pressure gradient, and no better explanation for the symptoms [2,8,16,29].

Endovascular stenting is often preferred as the secondary intervention because it avoids repeat open dissection. Columbo JA et al. described a multidisciplinary algorithm using initial laparoscopic release, followed by selective stenting and, rarely, bypass for persistent stenosis and symptoms [37]. This staged approach avoids unnecessary implantation in patients whose symptoms resolve after release alone.

Some centers have reported hybrid procedures combining laparoscopic release and intraoperative celiac stenting. Hybrid treatment is technically feasible, but routine stenting may overtreat patients who would improve after decompression alone, exposing them to antiplatelet therapy, restenosis, and device-related complications [38]. Therefore, it should remain selective rather than systematic.

Open reconstruction, including CA reimplantation, patch angioplasty, aortoceliac bypass, or aortohepatic bypass, may be appropriate for persistent occlusion, complex fixed lesions, failed endovascular treatment, or arterial injury [2,8,29]. These procedures should be concentrated in centers with mesenteric vascular expertise [16].

Treatment Outcomes

Across published studies, early partial or complete improvement is usually observed in 70-85% of selected patients, whereas durable freedom from failure is lower, commonly around 60-75% [22,27,29]. Recurrence may develop months or years later and can occur despite technically successful decompression and normal postoperative imaging [22,29]. Conversely, persistent radiographic stenosis does not necessarily indicate treatment failure.

Classical postprandial pain, weight loss, food avoidance, and a coherent symptom-imaging relationship appear to identify a more responsive phenotype [22]. A recent large single-surgeon analysis reported greater quality-of-life improvement when classical symptoms and supportive imaging were both present than when referral was based largely on imaging [39]. Vomiting, unprovoked pain, diffuse pain, opioid dependence, multiple functional disorders, and symptoms unrelated to eating or exertion have been inconsistently associated with poorer response. Age, sex, degree of stenosis, post-stenotic dilatation, expiratory velocity, and respiratory change have not demonstrated reproducible predictive value across studies [22,40]. Brody F et al. proposed a model incorporating age and baseline expiratory velocity, but external validation has been limited [40]. The absence of improvement after celiac plexus block may predict failure, whereas a strong positive response may enrich the likelihood of benefit [15,22]. Psychiatric comorbidity deserves nuanced interpretation. It may be associated with poorer quality-of-life outcomes, but it is neither proof that symptoms are non-organic nor an absolute contraindication to surgery [21]. Patients should receive appropriate mental health treatment and realistic counselling rather than categorical exclusion.

Major operative hazards include injury to the abdominal aorta, CA, common hepatic artery, splenic artery, left gastric artery, or inferior phrenic vessels. Bleeding is the principal cause of conversion from minimally invasive to open surgery [27]. Other reported complications include pneumothorax, pancreatitis, lymphatic leakage, splenic infarction, hepatic ischemia, arterial thrombosis or dissection, ileus, infection, and incisional hernia after open surgery [9,27,29]. Release may alter autonomic signaling and produce transient diarrhea, orthostatic symptoms, nausea, or altered gastric motility. Conversely, gastrointestinal dysmotility may improve when the celiac plexus is decompressed. Patients with substantial preoperative weight loss require attention to electrolytes, micronutrient deficiencies, and gradual restoration of oral intake [27,29].

Mortality is uncommon in contemporary high-volume series, but the possibility of major vascular hemorrhage means that treatment should not be regarded as minor surgery [16]. Appropriate vascular expertise, availability of blood products, and a clear conversion strategy are essential.

Postoperative Surveillance and Management of Persistent Symptoms

Clinical response should be assessed with standardized symptom and quality-of-life instruments rather than a binary question about improvement. Relevant outcomes include postprandial pain frequency and severity, ability to eat normally, weight change, analgesic use, return to work or education, exercise tolerance, and gastrointestinal symptoms [16,22,29].

Duplex USG can document changes in velocity and respiratory variation, but routine imaging findings must be interpreted in conjunction with symptoms [16,18,20]. Early postoperative improvement followed by recurrent symptoms should prompt evaluation for incomplete release, scar-related recompression, residual fixed stenosis, CA thrombosis, or an alternative diagnosis [22,27,37].

CTA or MRA is appropriate when symptoms persist or recur. If a significant residual lesion is present, catheter angiography with pressure-gradient assessment may help determine whether secondary stenting or reconstruction is justified [16,37]. In the absence of a hemodynamically important lesion, repeat vascular intervention is unlikely to help, and the diagnostic process should be reopened [20,22].

Reintervention may be considered when imaging demonstrates clear incomplete release or recurrent fibrous compression, and the symptom pattern remains typical [16,20,37]. Repeat neurolysis alone has uncertain durability [22,29]. Persistent symptoms with normalized anatomy should trigger reassessment for gastroparesis, biliary or pancreatic disease, functional gastrointestinal disorders, abdominal wall pain, autonomic dysfunction, and centralized pain [8,21,22].

Pediatric and Adolescent Patients

MALS is increasingly recognized in adolescents, often after prolonged investigation for abdominal pain, nausea, exercise intolerance, and weight loss. Pediatric series report postoperative improvement rates broadly similar to adult cohorts, but many patients have overlapping autonomic, gastrointestinal, or connective-tissue disorders [29].

Treatment principles are unchanged: exclude other disease, demonstrate dynamic compression, use multidisciplinary selection, and perform complete release with neurolysis. Particular care is needed to avoid attributing eating difficulties, anxiety, or functional pain automatically to MALS [29,41]. A large pediatric series reported low perioperative complication rates, but freedom from treatment failure was approximately 61%, again demonstrating that technically safe release does not guarantee durable symptom resolution [41].

Proposed Diagnostic and Therapeutic Algorithm

**Figure 1:**
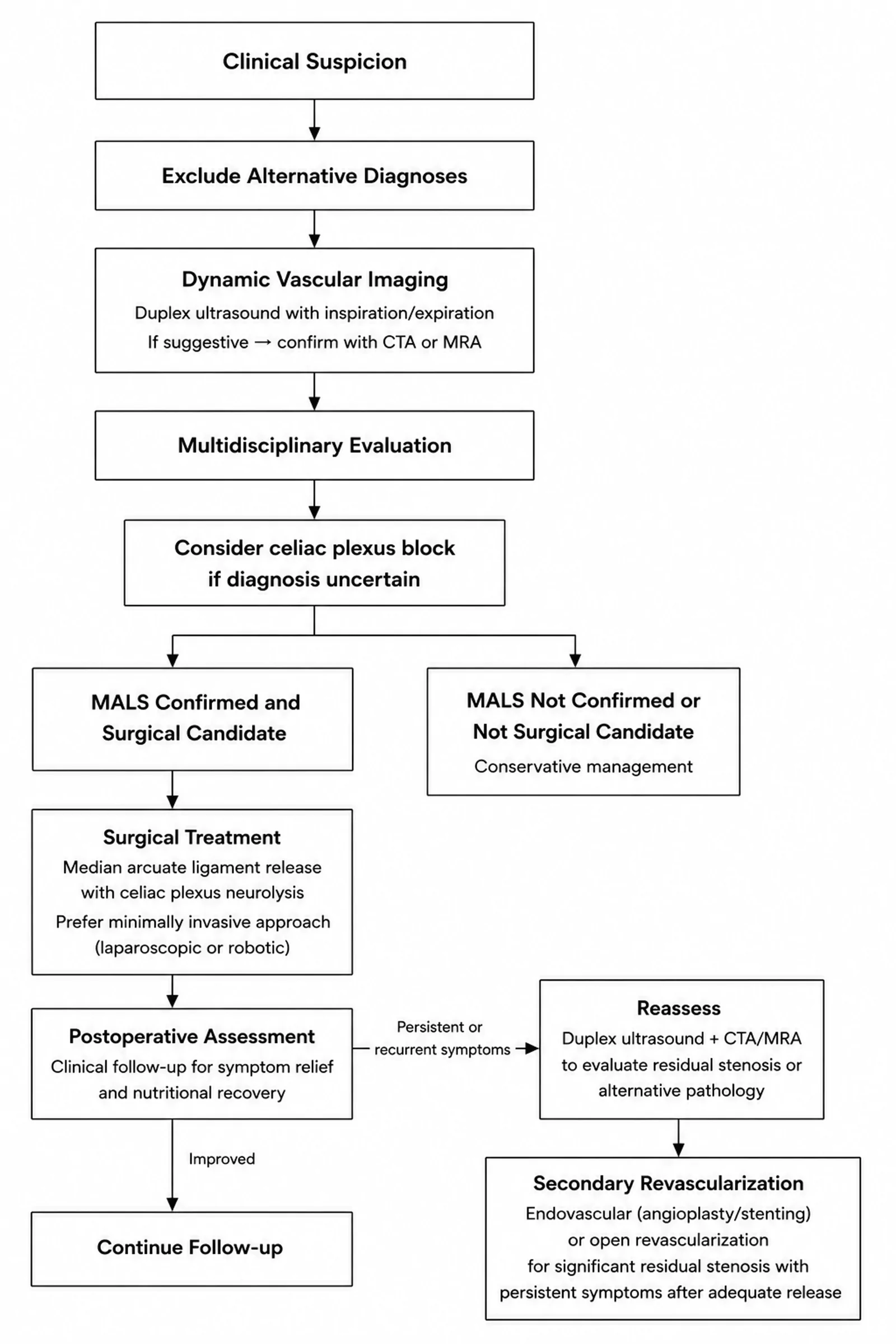
Proposed diagnostic and therapeutic algorithm for median arcuate ligament syndrome. CTA: Computed tomography angiography; MALS: Median arcuate ligament syndrome; MRA: Magnetic resonance angiography. Figure created by the authors. Image credit: Dimitrios A. Chatzelas

The available evidence supports a structured, multidisciplinary approach to the evaluation and management of patients with suspected MALS (Figure 1). Because CA compression is frequently an incidental anatomical finding, the diagnosis should only be considered in patients with compatible chronic symptoms after exclusion of more common gastrointestinal, hepatobiliary, pancreatic, metabolic, and vascular disorders. Dynamic duplex USG followed by inspiratory and expiratory CTA or MRA should confirm external CA compression before intervention is contemplated. Patients fulfilling clinical and imaging criteria should undergo multidisciplinary assessment, with celiac plexus blockade considered in selected cases with diagnostic uncertainty or a predominantly neurogenic presentation. Surgical MAL release with celiac plexus neurolysis remains the cornerstone of treatment, preferably using a minimally invasive approach when appropriate expertise is available. Postoperative management should be guided primarily by clinical response rather than imaging alone. Persistent or recurrent symptoms warrant reassessment with vascular imaging to exclude residual fixed stenosis or alternative pathology. Secondary endovascular or open CA revascularization should be reserved for carefully selected patients with persistent symptoms and objectively significant residual CA stenosis after adequate surgical decompression. This staged strategy minimizes unnecessary vascular interventions [16,29].

Discussion

The contemporary treatment paradigm for MALS is best described as selective decompression rather than correction of an imaging abnormality. The most important therapeutic decision is not whether to use open, laparoscopic, robotic, or retroperitoneal access, but whether the patient has a sufficiently coherent syndrome to justify intervention. Particular attention should be given to the distinction between correction of radiographic celiac compression and treatment of a clinically coherent syndrome, because this distinction appears to be the principal determinant of appropriate intervention and meaningful outcome assessment [16,22,29].

Evidence favors surgical ligament release with celiac neurolysis as first-line definitive treatment in carefully selected patients [16]. Minimally invasive release generally offers less perioperative morbidity and faster recovery than open surgery, but claims of superior symptom durability should be interpreted cautiously because open procedures are often reserved for more complex disease, previous treatment failure, or arterial reconstruction [22,27,29]. Robotic assistance may improve ergonomics but has not yet demonstrated a clinically meaningful advantage sufficient to justify a universal preference [30,31,33].

The treatment effect remains difficult to quantify. Reported improvement rates are influenced by variable diagnostic thresholds, retrospective selection, loss to follow-up, unblinded patient-reported outcomes, and inconsistent definitions of success. Some studies classify any improvement as success, whereas others require complete symptom resolution or freedom from further intervention. This heterogeneity explains why immediate response rates approaching 85% coexist with three-year freedom-from-failure rates closer to 60% [22,27,29].

The vascular-versus-neurogenic debate has direct therapeutic implications. If ischemia were the predominant mechanism, a stronger association would be expected between postoperative symptom relief and normalization of CA anatomy or hemodynamics. The imperfect relationship between imaging normalization and symptom response argues against a purely vascular model [6,20]. Conversely, evidence from celiac plexus blockade and extensive neurolysis supports a neurogenic component, but block studies are small and enriched by selection [15,22,23]. Current evidence is most compatible with a heterogeneous or mixed pathophysiology in which mechanical arterial compression, altered collateral hemodynamics, celiac plexus irritation, gastrointestinal dysmotility, and central pain processing may contribute in varying proportions among patients [6,8,13,16].

Primary CA stenting should be avoided because it treats only the arterial lumen while leaving the external compressive force intact. A staged strategy is more rational: decompression and neurolysis first, followed by reassessment, then selective revascularization for persistent symptoms accompanied by a physiologically significant residual lesion. This approach minimizes unnecessary implantation and acknowledges that many patients improve despite residual radiographic narrowing [16,22,35-38].

Among the most important ongoing studies is the CARoSO trial, which is comparing video-assisted retroperitoneal endoscopic release with a sham procedure in patients meeting multidisciplinary consensus criteria. Because symptoms are subjective and surgery has substantial contextual and placebo components, a sham-controlled design is uniquely valuable. Results should clarify whether release provides benefit beyond perioperative expectations and may identify which outcomes and eligibility criteria should be used in future studies [16]. Until such evidence becomes available, clinicians should communicate uncertainty explicitly and avoid presenting surgery as predictably curative.

Limitations and Future Directions

The current evidence base has important limitations. Most publications are retrospective series from highly specialized referral centers, with considerable potential for selection, referral, and publication bias. Diagnostic criteria, operative techniques, extent of neurolysis, and indications for adjunctive revascularization differ substantially among studies. Outcomes are commonly based on unblinded patient-reported symptom improvement, while definitions of partial response, complete response, recurrence, and treatment failure are inconsistent. Follow-up is frequently short or incomplete, and some institutional publications may contain overlapping populations. Consequently, reported success rates should not be interpreted as precise estimates of treatment efficacy or generalized to patients selected solely on the basis of imaging findings.

Future studies should adopt consensus definitions for diagnosis, technical success, partial response, complete response, recurrence, and treatment failure. They should report symptom-specific outcomes, validated quality-of-life measures, nutritional recovery, opioid use, reinterventions, and follow-up beyond three years. Prospective registries should record celiac plexus block response, psychiatric and autonomic comorbidities, extent of operative neurolysis, intraoperative hemodynamics, residual stenosis, and the rationale for secondary revascularization.

## Conclusions

MALS is a clinicoradiologic diagnosis requiring compatible symptoms, exclusion of alternative diagnoses, and confirmation of dynamic CA compression. Incidental radiologic compression should not be treated. Current evidence supports multidisciplinary evaluation at specialized centers and careful counselling regarding the substantial possibility of persistent or recurrent symptoms. The contemporary management of MALS should be based on phenotype-driven patient selection. Complete division of the MAL with extensive celiac plexus neurolysis is the principal treatment. Laparoscopic and robotic-assisted approaches are generally preferred when expertise is available. Open surgery remains important for complex anatomy, conversion, and concomitant arterial reconstruction. Primary CA angioplasty without ligament release should be avoided. Secondary endovascular or open revascularization should be reserved for persistent compatible symptoms and objectively important residual stenosis after adequate decompression. Standardized reporting, prospective registries, and controlled trials are essential to determine the true effectiveness of operative release and refine the selection of patients most likely to benefit from it.
